# Wireless EEG Recording of Audiogenic Seizure Activity in Freely Moving Krushinsky-Molodkina Rats

**DOI:** 10.3390/biomedicines12050946

**Published:** 2024-04-24

**Authors:** Sergey Krivopalov, Boris Yushkov, Alexey Sarapultsev

**Affiliations:** 1 Institute of Immunology and Physiology, Ural Branch of the Russian Academy of Science, 620049 Ekaterinburg, Russia; a.sarapultsev@gmail.com; 2 GAUZ SO Institute for Medical Cell Technologies, 620026 Ekaterinburg, Russia

**Keywords:** audiogenic epilepsy, behavioral neurology, ecological validity, free movement recording, Krushinsky-Molodkina rats, neurogenetics, seizure mechanisms, wireless electroencephalogram (EEG)

## Abstract

This study investigates audiogenic epilepsy in Krushinsky-Molodkina (KM) rats, questioning the efficacy of conventional EEG techniques in capturing seizures during animal restraint. Using a wireless EEG system that allows unrestricted movement, our aim was to gather ecologically valid data. Nine male KM rats, prone to audiogenic seizures, received implants of wireless EEG transmitters that target specific seizure-related brain regions. These regions included the inferior colliculus (IC), pontine reticular nucleus, oral part (PnO), ventrolateral periaqueductal gray (VLPAG), dorsal area of the secondary auditory cortex (AuD), and motor cortex (M1), facilitating seizure observation without movement constraints. Our findings indicate that targeted neural intervention via electrode implantation significantly reduced convulsive seizures in approximately half of the subjects, suggesting therapeutic potential. Furthermore, the amplitude of brain activity in the IC, PnO, and AuD upon audiogenic stimulus onset significantly influenced seizure severity and nature, highlighting these areas as pivotal for epileptic propagation. Severe cases exhibited dual waves of seizure generalization, indicative of intricate neural network interactions. Distinctive interplay between specific brain regions, disrupted during convulsive activity, suggests neural circuit reconfiguration in response to escalating seizure intensity. These discoveries challenge conventional methodologies, opening avenues for novel approaches in epilepsy research and therapeutic interventions.

## 1. Introduction

Epilepsy is a complex neurological disorder characterized by an enduring predisposition to generate seizures, which are recurrent paroxysmal events that induce stereotyped behavioral alterations [1,2]. These seizures are underpinned by distinct neural mechanisms, and their differential diagnosis often includes various clinical conditions involving transient alterations in awareness and/or behavior [3].

Genetic rat epilepsy models have been instrumental in elucidating the underlying mechanisms of the disorder. The Krushinsky-Molodkina (KM) inbred rat strain was the first among strains selected for audiogenic epilepsy and is of significance along with other inbred strains, such as the GAERS and WAG/Rij strains [4,5]. Furthermore, rats with clinically relevant mutations provide a crucial source of comparative data [6].

Although electroencephalogram (EEG) setups, both animal restrain [7] and wireless [8,9,10], have been used to record brain activity in these models, methodological limitations persist. Specifically, the requirement for animal immobilization during EEG recordings significantly compromises the ecological validity of acquired data [8,9,10,11,12]. Furthermore, the effects of immobilization stress on both brain activity and external behavioral manifestations of seizures have not been adequately addressed [12,13,14,15,16]. In cases where the experiment design includes EEG recording during seizures, the significance of technical limitations in existing models increases the manifold.

The aim of this study is to develop and introduce a wireless EEG recording system for Krushinsky-Molodkina rats, designed to allow free movement during recording sessions. This methodology is expected to not only enhance the reliability and validity of the data but also allow simultaneous measurements of neural activity and behavior, thus serving as a tool for elucidating causal relationships.

The study was carried out at the Institute of Immunology and Physiology of the Russian Academy of Sciences and aimed to investigate rodent models and broader applications in the research of human epilepsy [17].

## 2. Materials and Methods

### 2.1. Animal Husbandry and Ethical Approvals

This study used nine male Krushinsky-Molodkina rats, aged between 6 and 7 months and weighing approximately 250 ± 30 g [18,19,20,21]. Animals were acquired through a cooperative agreement with the Laboratory of Physiology and Genetics of Behavior, Chair of Higher Nervous Activity, Biology Department at Moscow State University, named after M.V. Lomonosov. Eligibility for inclusion in the study was determined by seizure susceptibility; only rats that exhibited a classical 4-point seizure after initial phonostimulation were selected [19,20,21].

Visual assessment of the severity of the seizures was based on the position of the body at the conclusion of the locomotor excitation phase: 1 point indicates no fall, with no clonic–tonic seizures developing; 2 points indicate a fall in the stomach, with a predominance of the clonic component of seizures; 3 points indicate a fall in the stomach with the body rolling to the side, showing a predominance of the tonic component of seizures in the front part of the body, stretching of the forelimbs in the caudal direction, and amplitude seizures of the hind limbs; 4 points indicate a fall in the stomach with rolling to the side, tonic seizures of the entire body, with all limbs stretching in the caudal direction.

The rats were housed in a vivarium under controlled conditions, including individual cages, a complete diet, ad libitum access to water, and a 12 h light–dark cycle. All experimental procedures adhered to the current legislation of the Russian Federation and the European Convention for the Protection of Vertebrate Animals Used for Experiments and Other Scientific Purposes (2007/526/EC). Ethical approval was secured from the IIP UrB RAS Bioethics Commission (Act No. 17.22.06.23).

### 2.2. Experimental Design and Surgical Procedures

The animals were implanted with stainless steel insulated sterile needle electrodes (length: 12 mm; diameter: 0.3 mm; conical working part: 0.4 mm). Electrode resistance was predetermined. Surgeries were performed using a Drive stereotactic system (Neurostar, Germany), and anesthesia was administered by inhalation of isoflurane. Intraoperative monitoring included ECG, spirometry, thermometry, and the assessment of unconditioned reflexes, such as the corneal reflex.

#### 2.2.1. Electrode Placement, Orientation and Stabilization

**Figure 1:**
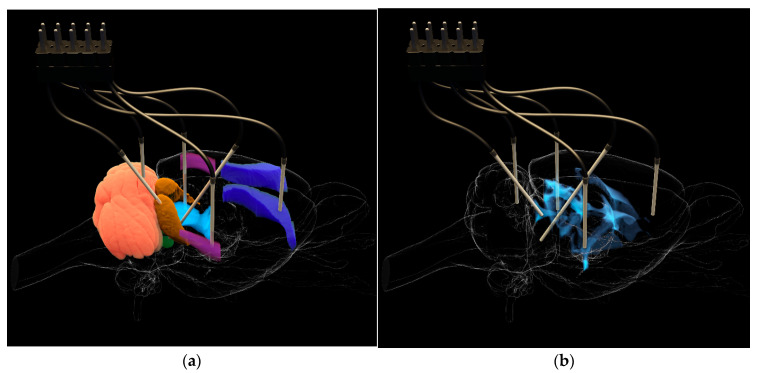
Electrode placement in the brain of KM rats: (**a**), relative to the investigated zones (EC IC—brown, VLPAG—light blue, PnO—green, AuD—violet, M1—dark blue) and cerebellum; (**b**), relative to the ventricular system.

The working parts of the electrodes were precisely positioned relative to the Bregma point, in accordance with established protocols [22]. Five working electrodes were introduced into each animal in specific brain zones responsible for the initiation, development, and external manifestations of audiogenic seizures (Figure 1). These included ECIC—external cortex of the inferior colliculus (2.0; −8.5; 4.0) of the brain, PnO—pontine reticular nucleus, oral part (1.2; −8.0; 8.0), AuD—dorsal area of the secondary auditory cortex (6.5; −3.1; 5.4), M1—motor cortex (3.7; −3.5; 2.5), and VLPAG—ventrolateral periaqueductal gray matter (−0.8; −7.8; 6.0). Furthermore, a reference electrode was implanted into the cortex of the fifth lobe of the cerebellum (0.0; −11.3; 4.0). Thus, four working electrodes were installed in the paired brain zones of the right hemisphere and one in an unpaired structure on the left. Brain atlas scaling, based on preliminary craniometry data, was used to improve the accuracy of electrode placement.

**Figure 2:**
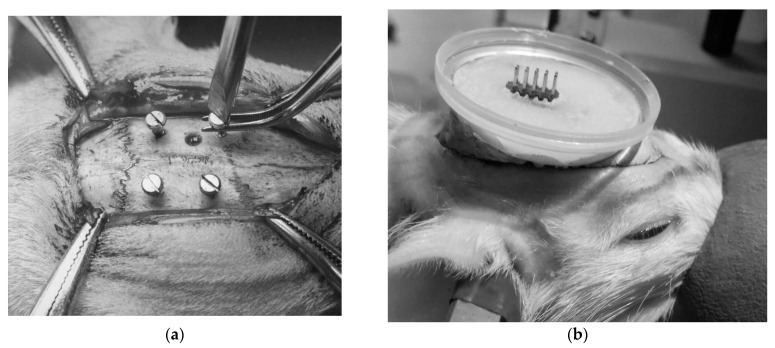
Stereotactic surgery stages: (**a**) installation of cortical screws; (**b**), fixation of the connector within the protective housing base.

Electrode angles were theoretically determined to minimize the distance to target zones while avoiding critical structures. Electrodes were introduced through 0.9 mm holes drilled into the skull at coordinates corresponding to the targeted brain regions. The electrodes were stabilized with fast-hardening epoxy materials and fortified with crystalline Nitrofural (furacilin). Four cortical screws (M1.5, stainless steel), positioned bilaterally in the frontal and parietal bones, served as anchor points and were connected to form a ground circuit (Figure 2).

#### 2.2.2. Transmitter and Connector Installation and Postoperative Care

A plastic protective housing was utilized to protect the transmitter and connector from potential damage during induced seizures. To facilitate reliable detachable electrical connections, a specialized adapter with enlarged connecting elements was utilized. A lighter alternative battery (LIR 2032 3.6 V) replaced the standard battery, reducing the transmitter weight by 2.5 g. This adjustment enabled the placement of the transmitting device within the protective housing above the transmitter, granting complete freedom of movement to the head and eliminating the need for separate body fixation.

**Figure 3:**
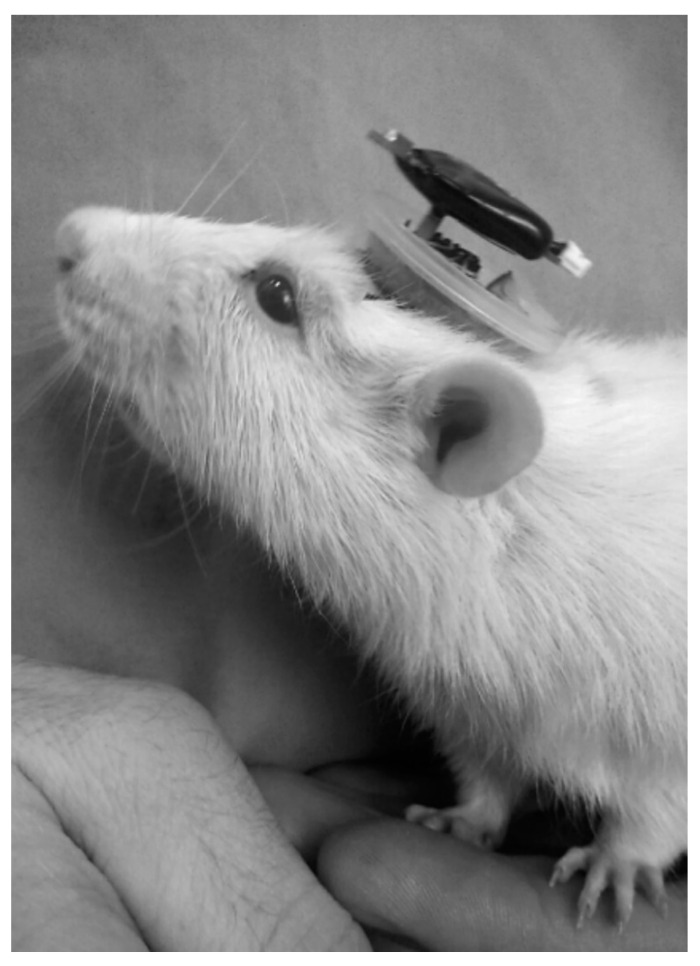
Appearance of the head-mounted transmitter in a rat during the preliminary stages of technique evaluation.

After surgical procedures, the animals were transferred to a vivarium for a two-week recovery period under veterinary supervision (Figure 3). Animals exhibiting symptoms such as wound suppuration, neurological depression, or refusal to eat or drink were immediately excluded from the experiment.

#### 2.2.3. EEG Recording and Signal Processing

EEG signals were captured using a multichannel digital polygraph, PowerLab 8/35—PL3508 (ADInstruments, Oxford, United Kingdom), paired with LabChart software v7.3.7 and a five-channel wireless transmitter and receiver (TBSI 5 Ch Wireless Neural Headstage—TB5653/F-LED; TBSI 5 Ch Wireless Neural Receiver—TB5663/FV). A total of 38 seizures in 9 animals were analyzed. LabChart and Neuron-Spectr.Net Omega v1.5.9.0 software facilitated the electroencephalogram processing, which included visual analysis, stage arrangement, amplitude measurement, and determination of characteristic frequency waves.

#### 2.2.4. Experimental Protocol

After recovery, animals were individually placed in a one-meter diameter “Open Field” arena (Open Science, Moscow, Russia) for concurrent EEG and behavioral recording. Background neural activity was recorded for the first ten minutes, followed by phonostimulation to provoke convulsive seizures. Audiogenic seizures were elicited using a multifrequency sound stimulus produced by an electric bell (sound pressure level at 1 m: 100 dB).

Seizure intensities were assessed according to the Krushinsky scale [18], with scores ranging from 1 (limited to motor excitation) to 4 (tonic and clonic convulsions with opisthotonus). We identified and coded several distinct stages of a four-point seizure, as described in our previous work [19].

To examine the potential effects of repetitive seizures on EEG activity, serial phonostimulations were performed at 15 min intervals, timed from the animal’s emergence from postictal cataleptoid state to the next phonostimulation. This process continued until the animals no longer responded to the auditory stimuli.

#### 2.2.5. Statistical Analysis

Data were analyzed in Statistica 10 software (v 10.0.1011.0), using nonparametric methods, including Friedman rank analysis of variance and the Mann–Whitney U test.

## 3. Results

### 3.1. Electroencephalographic Recordings

**Figure 4:**
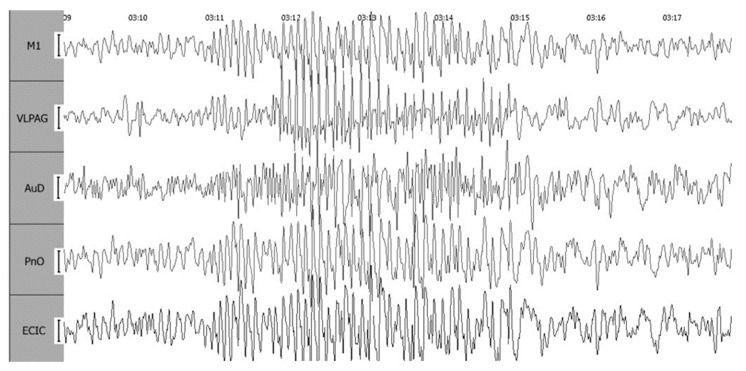
Sleep spindles during background recording. Leads include ECIC—external cortex of the inferior colliculus; PnO—pontine reticular nucleus, oral part; AuD—dorsal area of the secondary auditory cortex; M1—motor cortex; and VLPAG—ventrolateral periaqueductal gray matter. The calibration interval is set at 100 mV.

Electroencephalograms obtained using the described method are remarkably free of artifacts, allowing a clear visual assessment across all channels. Notably, sleep spindles are readily distinguishable against the background of stage 2 slow-wave sleep (Figure 4).

#### 3.1.1. Stages of 4-Point Seizures

**Figure 5:**
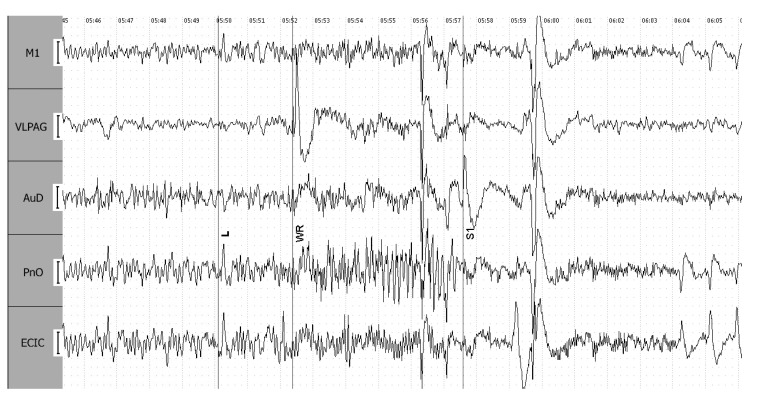
Initiation of a single wave 4-point epileptiform seizure, with clearly delineated stages. L—latency period, WR—locomotor excitation phase, S1—stage of clonic–tonic convulsions, with vertical lines marking the transitions between these stages as identified by external manifestations. Leads include ECIC—external cortex of the inferior colliculus; PnO—pontine reticular nucleus, oral part; AuD—dorsal area of the secondary auditory cortex; M1—motor cortex; and VLPAG—ventrolateral periaqueductal gray matter. The calibration interval is set at 100 mV.

EEGs facilitate the visual identification of the convulsive and postictal phases of a 4-point seizure, traditionally defined in an external observation. The latency (L) period demonstrates a notable increase in amplitude in the external cortex of the inferior colliculus (ECIC). During the locomotor excitation stage (WR), there is a marked increase in signal amplitude, which then decreases with the onset of clonic–tonic convulsions (S1). The convulsive period is distinguished by rhythmic peak–wave complexes in the ECIC, PnO, and M1 leads, which ultimately shift to synchronized oscillations of similar amplitude (Figure 5).

#### 3.1.2. Postoperative Effects

Electrode implantation influences the characteristics of seizures in experimental subjects. Initial postoperative stimulation uncovers the heterogeneity within the test group: merely 55.6% of the rats demonstrate a classic 4-point seizure. The rest exhibit milder convulsions, with some ceasing immediately after the WR stage, corresponding to a 1–2 point rating on the Krushinsky scale. In particular, no 3-point seizures were observed during the initial postoperative assessment.

#### 3.1.3. Amplitude Analysis

The observed decrease in seizure intensity after surgery requires a separate amplitude analysis for seizures of varying strengths and their subsequent postictal states. In this scenario, the background EEG assumes vital importance, as the neural response to auditory stimuli is significantly affected by the initial state of brain activity.

#### 3.1.4. Analysis of 4-Point Seizures

In cases of 4-point seizures, the highest amplitude recordings are noted in the ECIC. Lower amplitudes, approximately 80% of those in the ECIC, are observed in the PnO and AuD regions, whereas the remaining leads display voltage levels at about 45% of the ECIC amplitude. The L period is marked by a significant increase in amplitude (15–20% relative to baseline) in both the ECIC and PnO leads. During the WR stage, moderate increases in amplitude in the ECIC and VLPAG are observed, along with a sharp increase in PnO amplitude (to 140% of the values of the L stage values), with synchronized amplitude across the cerebral cortical areas. The S1 stage witnesses a sharp decline in amplitude across all leads (45–65% relative to baseline), followed by the stage of transient apnea (A), characterized by minimal signal amplitudes (15–25% of baseline).

Subsequent to stage A, another convulsive phase, the stage of clonic seizures (S3) is observed, where a 4-point seizure manifests itself as a significant increase in amplitude across all leads compared to the values of stage A, indicating at least two waves of generalization of seizures. However, this amplitude surge is brief, and the recorded values never return to baseline levels, instead staying within 50–75% of those values.

**Figure 6:**
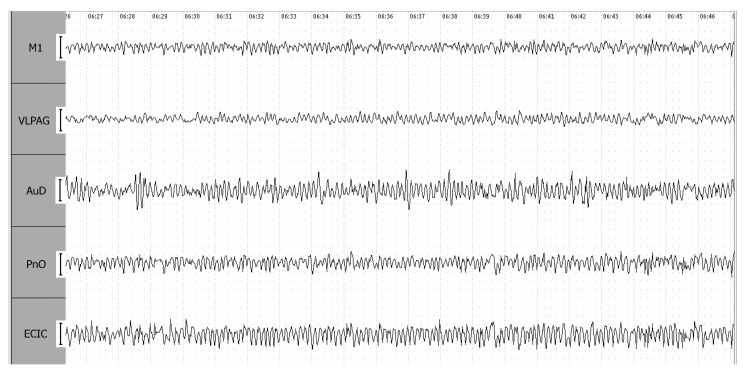
EEG during the breathing recovery stage (R1) of a 4-point seizure. Leads are as follows: EC IC—external cortex of the inferior colliculus; PnO—pontine reticular nucleus, oral part; AuD—dorsal area of the secondary auditory cortex; M1—motor cortex; VLPAG—ventrolateral periaqueductal gray matter. The calibration interval is set at 100 mV.

The re-establishment of a stable respiratory rhythm in the postictal breathing recovery stage (R1) of a 4-point seizure is accompanied by a further reduction in AuD amplitude to levels observed during the S1 stage, along with notable rhythm synchronization across all leads (Figure 6).

#### 3.1.5. Subsequent Stages

**Figure 7:**
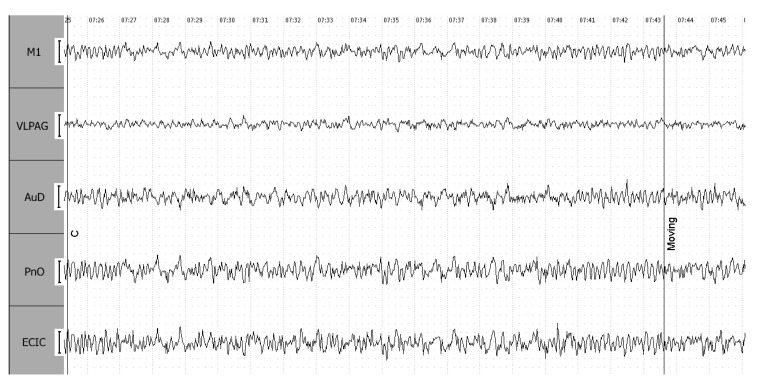
End-of-postictal cataleptoid state (C) in a 4-grade epileptiform seizure. Leads: EC IC—external cortex of the inferior colliculus of the brain; PnO—pontine reticular nucleus, oral part; AuD—dorsal area of the secondary auditory cortex; M1—motor cortex; VLPAG—ventrolateral periaqueductal gray matter. The calibration interval is 100 mV.

During the shortness-of-breath stage (R2), rhythm synchronization is maintained among the ECIC, PnO, and M1 leads, without significant changes in amplitude. The cataleptoid state (C), marking the conclusion of the postictal period, is characterized by a 40% increase in amplitude across all leads, except ECIC (Figure 7).

### 3.2. Electroencephalographic Characteristics across Seizure Types

#### 3.2.1. 4-Point vs. 2-Point Seizures

Compared to 4-point seizures, EEG profiles of 2-point seizures exhibit distinct amplitude patterns in various neural regions. In particular, lower background amplitudes are observed in the ECIC and PnO regions, whereas higher amplitudes are detected in the M1 region. This amplitude reduction trend between ECIC and PnO persists throughout the latency period and the locomotor excitation stage (WR), with a subsequent rise in amplitude observed in the VLPAG and AuD areas. As the clonic–tonic stage (S1) progresses, higher signal values compared to 4-point seizures are evident in all leads. Relatively elevated values in the VLPAG and M1 leads continue into the clonic seizure stage (S3). A nuanced pattern emerges during the breathing recovery stage (R1) and the cataleptoid state (C), characterized by varied amplitudes in the mentioned regions.

#### 3.2.2. One-Point Seizures

EEG amplitudes during 1-point seizures largely mirror those of 2-point seizures, albeit with some exceptions. For instance, during background recordings, higher amplitude values are observed in the AuD region instead of M1. Furthermore, the postictal period of 1-point seizures exhibits amplitudes that closely resemble background levels, raising questions about the occurrence of a cataleptoid state in these animals.

#### 3.2.3. Effects of Repeated Phonostimulation

A series of phonostimulations led to notable alterations in the characteristics of seizures. Initially, rats displaying 4-point seizures experienced a reduction in seizure intensity by the third or fourth phonostimulation, transitioning to 1-point seizures without progressing to the cataleptoid state. Subsequent phonostimulations initially intensified seizures to 2 points and induced a novel postictal state of hyperkinesis, eventually resulting in a complete loss of sensitivity to sound after 6–7 repetitions.

#### 3.2.4. Postictal Periods: Hyperkinesis versus Cataleptoid State

The phenomenon of myoclonic hyperkinesis, emerging upon repeated stimuli, as an alternative to the postictal cataleptic state (temporary loss of voluntary movements), entails abrupt singular muscle contractions of the torso and limbs.

These contractions are strong enough to cause the animal to engage in frequent low jumps. Pauses between contractions were barely sufficient for the animal to assume the correct sitting posture. The gradual weakening of seizure strength and frequency (from 2 Hz at the beginning of the phenomenon to 0.2 Hz at the end) was accompanied by the emergence of other locomotor behaviors: brief runs and short grooming acts (primarily face washing with the front paws).

Manifestations of myoclonic hyperkinesis, formed by a series of phonostimulations within a few hours, closely resemble those induced by kindling, not only externally but also on EEG. Characteristic peak–wave complexes are observed in the ECIC, as well as in the VLPAG and PnO, where they fade within a few seconds. Compared to postictal catalepsy, such hyperkinesis is characterized by lower amplitude values in the PnO region and higher values in the VLPAG, AuD, and M1 leads.

#### 3.2.5. Induced Hyperkinesis and Electrode Implantation

The phenomenon of induced hyperkinesis is notably absent in animals where electrode implantation reduced seizure strength to 1–2 points. In such cases, phonostimulation initially enhances the intensity of seizures temporarily, but by the third or fourth repetition, it ceases to elicit any response.

### 3.3. Correlation Analysis of Amplitude Characteristics

Despite these diverse effects, electroencephalograms of seizures with similar intensity displayed a high degree of similarity in amplitude characteristics across all leads and animals (Table A1 and Table A2). These findings support the potential use of cumulative statistical analyses, such as correlation analysis (provided formal requirements are met), to assess the interactive dynamics of the neural structures being studied.

#### 3.3.1. Correlation Analysis of EEG Signal Amplitudes across Neural Regions

An initial assessment of background amplitude correlations across different neural regions revealed two distinct blocks of highly correlated structures. Specifically, ECIC and PnO showed a very strong correlation (r = 0.98), as did AuD and M1 (r = 0.95). Furthermore, these latter structures exhibited a strong correlation with VLPAG (r = 0.86 and r = 0.81, respectively). In particular, an antagonistic relationship existed between these two blocks, characterized by negative correlation coefficients ranging from −0.55 to −0.74.

#### 3.3.2. Impact of Sound Stimulus

The introduction of an auditory stimulus did not alter the correlation relationships within the VLPAG, AuD, and M1 block. However, during the latency period, the negative correlation between ECIC on one side, and VLPAG and AuD intensified (r = −0.71 and r = −0.83, respectively), while the correlation between PnO and ECIC (r = 0.69), and PnO and M1 (r = −0.14) weakened.

Locomotor Excitation Stage (WR). This stage was characterized by the complete independence of amplitude in the ECIC from VLPAG, AuD, and PnO. Coupled with the re-establishment of a strong negative correlation between PnO and M1 (r = −0.83), against the backdrop of the integrity of the “block” VLPAG-AuD-M1, this observation suggests the potential development of a focal seizure.

Convulsive Stages (S1, S2). During these stages, all leads exhibited only positive correlations, both within and between blocks. These correlations, ranging from 0.64 to 0.95, occurred against a backdrop of a decrease in signal amplitude across all leads, indicating the generalization of a convulsive seizure.

Apnea Stage (A). The ECIC lead showed weak positive correlations, while negative correlations emerged between VLPAG on one side, and PnO, AuD, and M1 on the other (r = −0.71, r = −0.73, and r = −0.72, respectively). However, positive correlations between these latter structures remained robust.

Respiratory Recovery Stage. The onset of individual respiratory movements reinstated both intra-block and inter-block correlations, albeit at weaker levels compared to the background state.

Clonic Convulsions (S3). The correlation pattern at this stage mirrored that of S1 and S2, with coefficients greater than r = 0.83, which confirms the interpretation of observed clonic convulsions as a second wave of a generalized seizure.

Dyspnea and Postictal Cataleptoid State. During the dyspnea stage, antagonistic relationships between ECIC and the VLPAG-AuD-M1 block were re-established. PnO was positively correlated not only with ECIC (r = 0.43) but also with VLPAG and M1 (r = 0.43 and r = 0.21, respectively). At the same time, a weaker correlation than in the background was observed in the cortex between AuD and M1 (r = 0.57). The cataleptoid state in the postictal period demonstrated a landscape of correlation closely mirroring the background, with increased negative correlations between ECIC and PnO with VLPAG (r = −0.79), and reduced negative correlations with AuD and M1 (r = 0.60 and r = 0.55, respectively).

The observed correlation patterns between different neural areas and seizure stages, particularly in the context of a 4-point seizure, offer significant insights into the underlying neural dynamics. The data obtained from this study could serve as the basis for further exploration of the mechanisms of seizure onset, propagation, and generalization, as well as the development of related postictal states.

## 4. Discussion

Encephalographic studies in animals are pivotal for advancing our understanding of neural mechanisms. However, most animal studies use attached EEG systems and restrained conditions, which can complicate the correlation between EEG data and clinical manifestations of seizures [16,17,18,19,20,21,22,23]. Our introduction of a wireless EEG method for Krushinsky-Molodkina rats with genetically determined audiogenic seizures [20,21,22,23] has shed light on patterns that are unobservable with traditional methods.

Findings from our study facilitated the observation of new patterns in unrestrained animals, likely due to the absence of restraint-induced stress [9,10,11,12,13,14,15,16,24,25].

Therapeutic Implications of Electrode Implantation: Electrode implantation has been shown to reduce the clinical severity of seizures in a significant proportion of cases [26]. This is consistent with the literature on surgical treatment in epilepsy [27] and suggests a theranostic effect, where diagnostic procedures yield therapeutic benefits. The variation in impact between subjects may be influenced by brain lateralization and electrode placement asymmetry: electrodes were inserted into ECIC, VLPAG, AuD, and M1 on the right side and PnO on the left. Given that these structures are paired and the ratio of animals experiencing weakened seizures postsurgery is similar to the known ratio of “right-handed” and “left-handed” rats (50/50), there is reason to believe that brain lateralization modulates cognitive functions and seizure proneness.

Another possible reason for the reduction in the intensity of seizures after surgery, which does not exclude the aforementioned points, could be an immune reaction, whose role in epileptogenesis is actively being investigated [28,29,30]. This hypothesis aligns with our understanding of lateralization and could be developed to explore immune response heterogeneity in the brain.

Neurophysiological Factors: Background brain activity influences seizure strength and clinical presentation [31], with seizure propagation pathways remaining consistent between episodes. Hyperkinesis can be induced by a regimen of interrupted phonostimulations, providing the animals with additional recovery time after exiting the cataleptoid state [32]. This methodology appears to increase convulsive proneness in specific brain regions, enhancing seizure intensity in some animals [33]. Subsequent rapid nervous process depletion manifests in a prolonged postictal state, indicating deficient compensatory mechanisms after repeated seizures.

Individual Variability and Structural Roles: The study highlights individual variability in the exhaustion of the nervous process, which explains the absence of hyperkinesis in certain rats. Insufficient epileptic activity in the cortex or weakening in the inferior region may explain this variability. The EEG data reveal a dynamic interaction between the cortical and subcortical structures, with the signal amplitude in the inferior colliculus (Ic) and PnO reciprocally correlated with M1 and AuD. The Olivary complex (PnO) likely modulates the intensity of seizures, particularly when the divergence of amplitudes between the cortical and subcortical regions is maximized.

Interpretation of the Locomotor Stage: The locomotor excitation stage, characterized by EEG patterns and observable behavior, aligns more closely with background activity and latency than with the clonic–tonic seizure stages. As an isolated part of a seizure episode, it deserves further attention from the scientific community.

Overall, our wireless EEG approach provides a nuanced understanding of seizure mechanisms, offering valuable insights for future research and therapeutic interventions.

## 5. Conclusions

The implantation of electrodes in specific auditory and motor cortical regions, including Ic, PnO, VLPAG, AuD, and M1, attenuates convulsive seizures in approximately half of the KM rats studied. Furthermore, the amplitude characteristics of brain activity in the ECIC, PnO, and AuD regions at the time of stimulus presentation significantly influence the severity and nature of phonostimulation-induced seizures in KM rats. Seizures, ranging in strength from 1 to 4 points, predominantly originate in the inferior colliculi and serve as focal points for the propagation of epileptic activity. In severe 4-point seizures among KM rats, two waves of seizure generalization are evident: the S1–S2 phases are interrupted by a brief respiratory recovery phase (R1).

Within the context of epileptic activity, two stable reciprocal inter-relationships can be delineated: one between M1 and VLPAG and another between ECIC and PnO. These inter-relationships are temporarily disrupted during the generalization of convulsive activity. Induced hyperkinesis in KM rats can be achieved through a series of phonostimulations separated by intervals sufficient for the completion of the postictal cataleptoid state and the emergence of orienting–exploratory behavioral responses. The onset of induced hyperkinesis is associated with the activation of a secondary epileptic focus. Specifically, against a backdrop of high amplitude in the AuD region, the first structure to respond with increased amplitude to an auditory stimulus is M1. The remaining regions studied are only engaged during the locomotor excitation stage.

Locomotor excitation, in terms of both EEG amplitude characteristics and observable behavior, exhibits greater congruence with background activity, latency, and postictal catalepsy than with clonic–tonic convulsions. Therefore, this state should be considered an independent part of the audiogenic seizure. Lastly, the postictal cataleptic state in KM rats shows amplitude characteristics in all regions examined that closely resemble those preceding the initiation of the stimulus.

## 6. Study Limitations

The limitations of this study warrant meticulous delineation to offer a nuanced understanding of its results. A principal constraint is the small and homogeneous sample size of male Krushinsky-Molodkina rats, which hampers the study’s generalizability to other rat strains or, more broadly, to other animal models and human populations [34]. Furthermore, the study’s cross-sectional design restricts any longitudinal analysis, limiting insights into the temporal progression of seizure mechanisms or the long-term implications of electrode implantation.

Regarding the technology employed, while the wireless EEG system has its merits in enhancing data collection in unrestrained animals, it also poses questions concerning signal integrity, such as potential signal loss or interference. Importantly, during the method validation stage, the study included a comparative assessment of signals obtained through wired and wireless methods in the same animals. This comparison demonstrated complete equivalence in the recorded data and a reduced number of artifacts in the background recording.

The asymmetric placement of electrodes, particularly with PnO on the left side, could introduce biases in the assessment of the theranostic effects of electrode implantation. Furthermore, the study primarily focused on encephalographic data and did not adequately integrate other behavioral or physiological metrics that could have offered a comprehensive understanding of seizure activity.

The specific regimen of interrupted phonostimulations used in this study may have limited external validity, as it may not adequately represent natural, real-world stimuli inducing seizures. While our findings might be of some clinical relevance, particularly in terms of their theranostic implications, the translational applicability of these results to human clinical settings remains an open question. Concerns related to statistical power and operational definitions, such as the definitions used for seizure severity and intensity, also need to be carefully considered.

Lastly, although the study adhered to ethical guidelines, the ethical implications of animal studies are an ever-present consideration that should not be overlooked. By comprehensively discussing these limitations, the aim is to refine interpretations of the current findings and to guide the direction of subsequent research efforts.

In addressing the reviewer’s question regarding the rationale for specific electrode implantation sites: In selecting the implantation sites, we relied on extensive research documenting repeatedly validated findings by various researchers in the field of auditory physiology, as well as results from colleagues studying the phenomenology of audiogenic seizures in rodents. The anatomical position and size of the structures played a crucial role in choosing the implantation sites, with preference given to larger brain areas that could be accessed simultaneously from the dorsal surface of the skull. The main reason for selecting the dorsal part of the secondary auditory cortex over the primary cortex or the ventral area was its superior position, which allows easier surgical access during stereotaxic operations. Moreover, it is organizationally higher than the primary cortex, and if epileptic activity is detected in this area, it is also present in the primary cortex [35,36,37].

## Data Availability

The data supporting the findings of this study are available from the author upon request.

## Appendix A

**Table A1:** Amplitude correlation coefficients calculated between leads for different seizure stages.

Stage	IC	PnO	VLPAG	AuD	M1	Lead
B	1	0.98	−0.55	−0.64	−0.69	ECIC
	0.98	1	−0.57	−0.71	−0.74	PnO
	−0.55	−0.57	1	0.86	0.81	PAG
	−0.64	−0.71	0.86	1	0.95	AuD
	−0.69	−0.74	0.81	0.95	1	M1
L	1	0.69	−0.71	−0.83	−0.62	ECIC
	0.69	1	−0.69	−0.52	−0.14	PnO
	−0.71	−0.69	1	0.83	0.76	PAG
	−0.83	−0.52	0.83	1	0.81	AuD
	−0.62	−0.14	0.76	0.81	1	M1
WR	1	0.05	0.1	−0.07	0.33	ECIC
	0.05	1	−0.93	−0.6	−0.83	PnO
	0.1	−0.93	1	0.71	0.9	PAG
	−0.07	−0.6	0.71	1	0.76	AuD
	0.33	−0.83	0.9	0.76	1	M1
S1	1	0.86	0.79	0.81	0.86	ECIC
	0.86	1	0.93	0.76	0.71	PnO
	0.79	0.93	1	0.74	0.64	PAG
	0.81	0.76	0.74	1	0.95	AuD
	0.86	0.71	0.64	0.95	1	M1
S2	1	1	1	1	1	ECIC
	1	1	1	1	1	PnO
	1	1	1	1	1	PAG
	1	1	1	1	1	AuD
	1	1	1	1	1	M1
A	1	0.23	0.43	−0.13	0.24	ECIC
	0.23	1	−0.71	0.62	1	PnO
	0.43	−0.71	1	−0.73	−0.72	PAG
	−0.13	0.62	−0.73	1	0.62	AuD
	0.24	1	−0.72	0.62	1	M1
R1	1	0.74	−0.24	−0.57	−0.52	ECIC
	0.74	1	−0.21	−0.69	−0.31	PnO
	−0.24	−0.21	1	0.57	0.76	PAG
	−0.52	−0.31	0.76	0.76	1	AuD
	−0.57	−0.69	0.57	1	0.76	M1
S3	1	0.93	0.98	0.9	1	ECIC
	0.93	1	0.9	0.83	0.93	PnO
	0.98	0.9	1	0.93	0.98	PAG
	0.9	0.83	0.93	1	0.9	AuD
	1	0.93	0.98	0.9	1	M1
R2	1	0.43	−0.52	−0.74	−0.74	ECIC
	0.43	1	0.43	−0.17	0.21	PnO
	−0.52	0.43	1	0.79	0.79	PAG
	−0.74	−0.17	0.79	1	0.57	AuD
	−0.74	0.21	0.79	0.57	1	M1
C	1	1	−0.79	−0.6	−0.55	ECIC
	1	1	−0.79	−0.6	−0.55	PnO
	−0.79	−0.79	1	0.83	0.8	PAG
	−0.6	−0.6	0.83	1	0.9	AuD
	−0.55	−0.55	0.8	0.9	1	M1

**Note:** The symbols ECIC (external cortex of the caudal colliculi), VLPAG (ventrolateral periaqueductal gray), PnO (olivary complex), and AuD (dorsal area of the secondary auditory cortex), M1 (motor cortex) denote specific brain zones were electrodes were introduced.

**Table A2:** Average amplitude of background EEGs in animals with different seizure strengths, mV.

Stage or Period	4-Point Seizures	2-Point Seizures	1-Point Seizures	Hyperkinesis	Lead
B	36.27 ± 0.34	24.95 ± 0.33 ^4^	23.21 ± 1.29 ^4^	22.96 ± 0.07 ^4 2^	ECIC
	29.43 ± 0.40 ^I^	20.83 ± 0.47 ^I 4^	19.73 ± 1.22 ^4^	17.21 ± 0.56 ^I 4 2^	PnO
	15.41 ± 0.24 ^I P^	18.98 ± 2.05 ^I^	17.31 ± 1.39 ^I^	22.97 ± 1.08 ^4 1^	VLPAG
	28.45 ± 0.30 ^I V^	28.99 ± 3.10 ^P V^	22.32 ± 2.03 ^4^	43.10 ± 0.28 ^I P V 4 2 1^	AuD
	17.72 ± 0.27 ^I P V A^	22.63 ± 1.49 ^4^	18.51 ± 0.85 ^I^	23.99 ± 0.60 ^P A 4 1^	M1
L	43.44 ± 0.32	30.99 ± 1.65 ^4^	28.55 ± 2.32 ^4^	22.22 ± 0.34 ^4 2 1^	ECIC
	33.57 ± 2.13 ^I^	22.66 ± 1.07 ^I 4^	22.08 ± 1.55 ^4^	17.80 ± 0.68 ^I 4 2 1^	PnO
	15.29 ± 0.74 ^I P^	19.56 ± 1.85 ^I 4^	19.48 ± 1.62 ^I 4^	25.43 ± 1.40 ^P 4 2 1^	VLPAG
	27.51 ± 3.86 ^I V^	28.73 ± 2.80 ^V^	26.91 ± 2.65	43.13 ± 1.11 ^I P V 4 2 1^	AuD
	21.23 ± 2.45 ^I P^	24.03 ± 1.46 ^I^	20.91 ± 0.92 ^I^	38.03 ± 2.14 ^I P V 4 2 1^	M1
WR	47.75 ± 1.96	38.59 ± 0.89 ^4^	41.92 ± 3.63	45.37 ± 1.53 ^2^	ECIC
	88.13 ± 1.45 ^I^	40.43 ± 1.52 ^4^	54.27 ± 4.58 ^4 2^	32.46 ± 0.80 ^I 4 2 1^	PnO
	22.33 ± 1.81 ^I P^	36.05 ± 3.94 ^4^	28.45 ± 2.05 ^I P^	47.19 ± 1.24 ^P 4 2 1^	VLPAG
	25.58 ± 1.72 ^I P^	49.05 ± 4.62 ^4^	33.08 ± 3.45 ^P 2^	63.74 ± 2.07 ^I P V 4 2 1^	AuD
	23.10 ± 1.14 ^I P^	40.24 ± 2.72 ^4^	31.10 ± 2.20 ^P 4^	61.98 ± 1.86 ^I P V 4 2 1^	M1
S1	19.56 ± 0.21	29.15 ± 2.29 ^4^		28.10 ± 0.68 ^4^	ECIC
	12.54 ± 0.10 ^I^	29.26 ± 2.43 ^4^		20.80 ± 0.22 ^I 4 2^	PnO
	5.89 ± 0.33 ^I P^	29.15 ± 2.05 ^4^		30.95 ± 0.42 ^I P 4^	VLPAG
	15.07 ± 0.38 ^I P V^	36.90 ± 4.32 ^4^		51.31 ± 3.87 ^I P V 4^	AuD
	9.28 ± 0.65 ^I P V A^	25.47 ± 1.42 ^A 4^		40.15 ± 3.14 ^I P V 4 2^	M1
S2		23.74 ± 1.43			ECIC
		22.57 ± 0.90			PnO
		20.32 ± 2.22			VLPAG
		31.79 ± 4.19			AuD
		19.63 ± 1.62 ^A^			M1
A	4.26 ± 1.92	3.66 ± 1.61			ECIC
	3.95 ± 0.80	3.24 ± 1.43			PnO
	5.15 ± 0.94	5.53 ± 2.44			VLPAG
	6.85 ± 0.48 ^P^	5.09 ± 2.24			AuD
	3.62 ± 1.29	3.71 ± 1.64			M1
R1	21.59 ± 0.59	8.1 ± 2.07 ^4^			ECIC
	14.09 ± 0.67 ^I^	7.36 ± 1.88 ^4^			PnO
	6.57 ± 0.72 ^I P^	8.38 ± 2.6			VLPAG
	15.95 ± 2.26 ^I V^	8.29 ± 2.41			AuD
	7.39 ± 1.81 ^I P A^	7.63 ± 2.09			M1
S3	24.64 ± 2.06	21.23 ± 0.60			ECIC
	18.44 ± 2.43	17.47 ± 0.32 ^I^			PnO
	9.60 ± 0.88 ^I P^	16.35 ± 2.00 ^4^			VLPAG
	21.05 ± 0.64 ^V^	27.18 ± 2.97 ^P^			AuD
	10.99 ± 1.90 ^I A^	16.70 ± 1.01 ^I A 4^			M1
R2	21.75 ± 0.18				ECIC
	14.63 ± 1.17 ^I^				PnO
	6.41 ± 0.29 ^I P^				VLPAG
	12.2 ± 0.27 ^I V^				AuD
	8.62 ± 0.68 ^I P V A^				M1
PP	24.72 ± 1.63	19.17 ± 0.59 ^4^	23.06 ± 1.30 ^1^	22.09 ± 0.43 ^2^	ECIC
	19.87 ± 1.65	17.37 ± 0.76	19.45 ± 1.35	15.61 ± 0.31 ^I 4 2^	PnO
	9.22 ± 1.35 ^I P^	15.61 ± 1.25 ^4^	18.02 ± 1.47 ^4^	21.75 ± 0.29 ^P 4 2 1^	VLPAG
	17.05 ± 2.98	26.25 ± 3.15 ^I P V^	18.56 ± 1.08	46.94 ± 1.24 ^I P V 4 2 1^	AuD
	11.99 ± 1.17 ^I P^	17.89 ± 1.11 ^A 4^	18.65 ± 1.05 ^4^	32.29 ± 2.17 ^I P V A 4 2 1^	M1

**Note:** The symbols ECIC (external cortex of the caudal colliculi), VLPAG (ventrolateral periaqueductal gray), PnO (olivary complex), and AuD (dorsal area of the secondary auditory cortex), M1 (motor cortex) denote specific brain zones were electrodes were introduced. Indices 1, 2, and 4 in suprecript indicate significant differences from seizures of corresponding intensity. Letters I, P, V, and A in suprecript are the abbreviation of leads (I—ECIC, V—VLPAG, P—PnO, and A—AuD).
